# Selective Serotonin Reuptake Inhibitors for Treating Neurocognitive and Neuropsychiatric Disorders Following Traumatic Brain Injury: An Evaluation of Current Evidence

**DOI:** 10.3390/brainsci7080093

**Published:** 2017-07-25

**Authors:** John K. Yue, John F. Burke, Pavan S. Upadhyayula, Ethan A. Winkler, Hansen Deng, Caitlin K. Robinson, Romain Pirracchio, Catherine G. Suen, Sourabh Sharma, Adam R. Ferguson, Laura B. Ngwenya, Murray B. Stein, Geoffrey T. Manley, Phiroz E. Tarapore

**Affiliations:** 1 Department of Neurological Surgery, University of California, San Francisco, 1001 Potrero Avenue, Building 1, Room 101, San Francisco, CA 94110, USA; yuej@neurosurg.ucsf.edu (J.K.Y.); john.burke@ucsf.edu (J.F.B.); pavan8632@gmail.com (P.S.U.); ethan.winkler@ucsf.edu (E.A.W.); hansen.deng@ucsf.edu (H.D.); caitlinkrobinson1@gmail.com (C.K.R.); catherinegsuen@gmail.com (C.G.S.); s.sharm.01@gmail.com (S.S.); adam.ferguson@ucsf.edu (A.R.F.); manleyg@neurosurg.ucsf.edu (G.T.M.); 2 Brain and Spinal Injury Center, Zuckerberg San Francisco General Hospital, San Francisco, CA 94110, USA; 3 Department of Psychiatry, University of California, San Diego, CA 92093, USA; mstein@ucsd.edu; 4 Department of Anesthesia and Perioperative Care, University of California, San Francisco, San Francisco, CA 94143, USA; romain.pirracchio@ucsf.edu; 5 Department of Neurology, University of Utah School of Medicine, Salt Lake, UT 84112, USA; 6 Stritch School of Medicine, Loyola University Chicago, Chicago, IL 60660, USA; 7 San Francisco Veterans Affairs Medical Center, San Francisco, CA 94121, USA; 8 Department of Neurological Surgery, University of Cincinnati, Cincinnati, OH 45220, USA; ngwenyla@ucmail.uc.edu; 9 Department of Family and Preventive Medicine, University of California, San Diego, La Jolla, CA 92093, USA

**Keywords:** cognition, depression, meta-analysis, postconcussive disorder, selective serotonin reuptake inhibitor, sleep disturbance, traumatic brain injury

## Abstract

The prevalence of neuropsychiatric disorders following traumatic brain injury (TBI) is 20%–50%, and disorders of mood and cognition may remain even after recovery of neurologic function is achieved. Selective serotonin reuptake inhibitors (SSRI) block the reuptake of serotonin in presynaptic cells to lead to increased serotonergic activity in the synaptic cleft, constituting first-line treatment for a variety of neurocognitive and neuropsychiatric disorders. This review investigates the utility of SSRIs in treating post-TBI disorders. In total, 37 unique reports were consolidated from the Cochrane Central Register and PubMed (eight randomized-controlled trials (RCTs), nine open-label studies, 11 case reports, nine review articles). SSRIs are associated with improvement of depressive but not cognitive symptoms. Pooled analysis using the Hamilton Depression Rating Scale demonstrate a significant mean decrease of depression severity following sertraline compared to placebo—a result supported by several other RCTs with similar endpoints. Evidence from smaller studies demonstrates mood improvement following SSRI administration with absent or negative effects on cognitive and functional recovery. Notably, studies on SSRI treatment effects for post-traumatic stress disorder after TBI remain absent, and this represents an important direction of future research. Furthermore, placebo-controlled studies with extended follow-up periods and concurrent biomarker, neuroimaging and behavioral data are necessary to delineate the attributable pharmacological effects of SSRIs in the TBI population.

## 1. Introduction

Traumatic brain injury (TBI) is a critical public health concern recognized by the World Health Organization (WHO) as a leading cause of death and permanent disability worldwide at 10 million persons annually [1]. TBI contributes to approximately one-third (50,000) of all injury-related deaths in the United States (U.S.) each year, and 80,000 persons are discharged with TBI-related impairments [2,3]. While the incidence is 200 per 100,000 persons per year based on emergency department (ED) admissions, the rate of mild TBI is underestimated and severe TBI is overestimated [4].

In 1993 the American Congress for Rehabilitation Medicine (ACRM) defined mild TBI as loss of consciousness (LOC) <30 min, Glasgow Coma Scale (GCS) score of 13–15, and posttraumatic amnesia (PTA) <24 h [5]. Studies have shown that 10% of mild TBI patients continue to suffer from clinical symptoms at one year post injury [6,7]. Approximately 3.2 million persons in the U.S. live with long-term TBI-related disability [8]. Disorders of mood, arousal, attention, anxiety, and sleep disturbances are widely reported following TBI, presenting during both the initial stages of injury and the post-acute continuum of recovery. Psychiatric and emotional sequelae can persist even after neurologic function returns. While the true prevalence of these disorders is difficult to determine due to selection/reporting biases, the correlation between TBI and the development of neuropsychiatric disorders is well documented at 40%–48% and 23%–29% respectively [9,10]. These post-TBI sequelae in inpatient and outpatient settings remain difficult to manage clinically, exacerbating functional disability, perception of impairment, and effectiveness of rehabilitation.

One unifying mechanism underlying acquired neuropsychiatric conditions following TBI is disruption of the serotonergic system. Serotonin is an indolamine neurotransmitter with cell bodies located primarily in the caudal raphe nuclei of the medulla with projections to the cerebellum and spinal cord, and the rostral raphe nuclei in the pons supplying the prefrontal cortex, forebrain, limbic-striatal system, hippocampus, and cerebellum [11]. Genetic polymorphisms in coding regions for serotonin receptors and transporters have been implicated in depression, drug abuse, schizophrenia, aggression, and Alzheimer’s disease [12,13]. As serotonin cannot cross the blood-brain barrier, synthesis must occur de novo in the brain. During TBI the shearing of brainstem axons effectively disrupts pontine and medullary serotonergic projections, resulting in the decreased production and metabolism of serotonin [14,15,16,17].

Selective serotonin reuptake inhibitors (SSRIs) are a class of antidepressant agents that inhibit the reuptake of serotonin by monoamine transporters in the presynaptic cell, allowing for increased availability of serotonin in the synaptic cleft and increased/repeated stimulation of serotonergic postsynaptic receptors, leading to increased synaptic signaling. SSRIs are indicated for the treatment of psychiatric disorders including depression, obsessive-compulsive disorder, bulimia, and panic disorder through the modulation of neuronal cell survival and neuroplasticity. SSRIs increase extracellular serotonin (5-HT), activating the seven types of 5-HT receptors and many subtypes of each class, which can also be directly stimulated by the SSRI. Biomolecular benefits are the upregulation of transcription factor cAMP-responsive element binding protein (CREB) [18], brain-derived neurotrophic factor (BDNF) production [19], astrocyte glycogenolysis for energy supply [20], and inhibition of neurotoxicity and apoptosis [21,22]. In the management of neurodegenerative diseases—Alzheimer’s disease, Parkinson’s disease, stroke, Huntington’s disease, multiple sclerosis, and epilepsy—the benefits of SSRIs have been reported in small clinical studies. In the larger TBI population, irreversible damage to neurons result in substantial psychosocial dysfunction and post-concussive symptomatology, which in theory can be modulated by SSRIs through optimization of the neuronal metabolism and neurotrophic factors. A hallmark antidepressant effect begins at three to four weeks following administration and adherence is recommended for at minimum three to six months [23].

Serotonin modulates mood, arousal, emotion, and working memory among other states and/or symptomatologies, and therefore SSRIs constitute an attractive, titratable, and potentially long-term pharmacological intervention for post-TBI neurocognitive and neuropsychiatric deficits. Animal models have demonstrated the effectiveness of SSRIs in treating a range of neurological disorders [24]. In humans, evidence-based knowledge of the optimal management of psychiatric impairment —both temporary and permanent—in neurodegenerative disorders and following TBI have mainly emphasized upon reducing depression symptomatology with promising results. However, the management on other common neuropsychiatric disorders following TBI has been a challenge to investigate and exist in separate reports. In the current article, we comprehensively review and evaluate the evidence to date on SSRI treatment effects for the spectrum of post-TBI outcome domains including depression, cognition, post-concussive symptoms/syndrome, sleep disturbance, anxiety, and post-traumatic stress disorder (PTSD).

## 2. Methods

### 2.1. Study Selection

The initial literature search was performed using the Cochrane Central Register for Controlled Trials (CENTRAL) with the following search criteria: [“traumatic brain injury (ti, ab, kw)” and ((“selective serotonin reuptake inhibitor (ti, ab, kw)” or “citalopram (ti, ab, kw)” or “escitalopram (ti, ab, kw)” or “fluvoxamine (ti, ab, kw)” or “fluoxetine (ti, ab, kw)” or “paroxetine (ti, ab, kw)” or “sertraline (ti, ab, kw)”))] through 15 September 2016. A total of 13 original studies were identified. A second literature search was performed using the National Library of Medicine (NLM) PubMed database for all English language manuscripts matching the following criteria: [(traumatic brain injury [title/abstract, MESH terms]) and ((selective serotonin reuptake inhibitor [title/abstract, MESH terms] or citalopram [title/abstract, MESH terms] or escitalopram[title/abstract, MESH terms] or fluvoxamine [title/abstract, MESH terms] or fluoxetine[title/abstract, MESH terms] or paroxetine[title/abstract, MESH terms] or sertraline[title/abstract, MESH terms]))]. A total of 83 manuscripts were identified from NLM PubMed, of which seven manuscripts overlapped with CENTRAL search.

Three study authors (J.K.Y., J.F.B., P.S.U.) independently reviewed each article and its accompanying references for scientific merit, with focus on use of SSRI as a therapeutic intervention after TBI, and reached consensus regarding the inclusion of each reference into the current review. Any disagreements were adjudicated independently by the senior author (P.E.T.).

**Figure 1:**
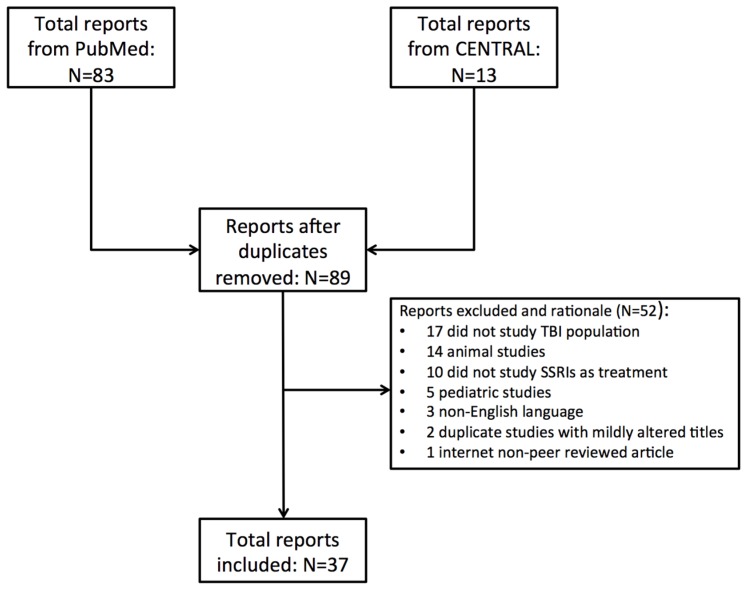
Flowchart of included studies. CENTRAL, Cochrane Central Register for Controlled Trials; SSRI, selective serotonin reuptake inhibitor; TBI, traumatic brain injury.

Of the 89 unique articles identified, 52 were removed due to lack of applicability to the current review (17 did not define TBI as the study population of interest, 14 were animal studies, 10 did not discuss SSRI as a treatment modality, five were pediatric studies, three were non-English language, two were repeated studies, and one was an internet article). A final total of 37 manuscripts (eight randomized-controlled trials (RCT), nine open-label studies, 11 case reports, nine review articles) were deemed fit for inclusion as part of the current review (Figure 1; Table 1).

Finally, manuscripts were assessed for the common endpoint of depression. The Hamilton Depression Rating Scale (Ham-D) [25] was the only common outcome measure across multiple studies and was therefore selected as the endpoint for our meta-analysis.

### 2.2. Statistical Analysis

For studies included in the meta-analysis, standard deviations and mean Ham-D values before and after intervention for both placebo and sertraline groups were recorded. Within each treatment group (sertraline, placebo) mean differences in Ham-D score was calculated using m_pre_ − m_post_ and pooled standard deviations of the standard mean difference was calculated using sqrt(((*n* − 1) × SD_pre_ + (*n* − 1) × SD_post_) ÷ (2*n* + 2)) , where m_pre_ , SD_pre_ are the mean and standard deviation pre-intervention and m_post_, SD_post_ are the mean and standard deviation post-treatment. The standardized mean difference, pooled standard deviation and sample size values for the intervention and placebo groups of each study were entered into the *R* statistical package MAVIS (Meta-Analysis via Shiny) R-Shiny application [62]. Hedge’s *g*, a standardized measure of the number of standard deviations the control and treatment means differ by, is reported for the meta-analysis as an unbiased measure of effect size [63].

## 3. Results

### 3.1. Depression

#### 3.1.1. Background and Pathophysiology

Serotonergic neuronal projections modulate mood and play a central role in depression symptomatology [64]. Jarring movement from traumatic injury often causes the brain to strike against the anterior fossae leading to damage of anterior frontal lobe neuronal networks, which are heavily innervated by serotonergic projections from the raphe nuclei [65]. Most neurotransmitter concentrations normalize within a matter of days but chronic deficits in serotonergic, dopaminergic, cholinergic and adrenergic systems cause persistent neuropsychiatric sequelae. The incidence for post-TBI depression ranges from 6% to 77% due to variable diagnostic standards [56], and up to 53% in the first year post injury [66]. Standard treatments include pharmacotherapy, of which SSRIs are first-line, and cognitive behavioral therapy (not included in the current review) [59]. Consequently, judicious use of SSRIs in the management of post-TBI depression is of high importance and impact.

#### 3.1.2. Evidence Synthesis: Sertraline

Sertraline is the most-often investigated SSRI in post-TBI depression, possibly due to improved understanding of its interaction with cytochrome P450 (CYP450) enzymes for metabolism compared to other SSRIs [59]. Five RCTs, two open-label studies, and two case reports investigate the use of sertraline to treat depression.

Regarding the potential of early prophylaxis for depression, e.g., the reduction of depression incidence and/or symptoms from administration of SSRI relatively soon after suffering TBI, Novack and colleagues randomized 99 moderate to severe TBI patients without baseline antidepressant use or drug abuse to sertraline 50 mg/day (*n =* 49) vs. placebo (*n =* 50) for one year. Eligible patients were enrolled no more than eight weeks following TBI, and treatment (sertraline vs. placebo) was started on the day after enrollment. No sertraline-treated patients developed depression assessed by the Ham-D Short Form. However, patients on sertraline scored poorer on the depression subscale of the Neurobehavioral Functioning Inventory (NFI), which is an overall an 83-item self-report inventory quantifying symptoms and behaviors associated with acquired neurological injury across six domains (depression, somatic complaints, memory/attention difficulties, communication deficits, aggressive behaviors, motor impairment), compared to placebo at three months. No significant differences were found between three-month and 12-month scores for either Ham-D or NFI [32].

Another large recent double-blind RCT performed by Jorge and colleagues attempts to address the utility of sertraline for prophylaxis following TBI. In 94 adult patients suffering mild to severe TBI without ongoing baseline depression or mood disorders, and with complete recovery of posttraumatic amnesia within four weeks of injury, were administered sertraline or placebo at the dose of 25 mg/day for the first five days, 50 mg/day for the next five days, and 100 mg/day thereafter [29]. Diagnosis of depression was performed using the Mini-International Neuropsychiatric Interview and the Diagnostic and Statistical Manual for Mental Disorders, Fourth Edition (DSM-IV) criteria at 2-, 4-, 8-, 12-, 16-, 20- and 24-week in-person follow-up visits, as well as telephone visits at weeks 6, 10, 14, 18, and 22. The authors found that the number needed to treat (NNT) to prevent depression after TBI at 24-weeks was 5.9 persons (e.g., on average, 5.9 patients will need to undergo treatment for one case of depression to be prevented). Sertraline patients were more likely to experience dry mouth and diarrhea, however neither sexual side effects nor suicidal ideation were attributable to sertraline administration [29].

Ashman and colleagues conducted a 10-week double blind RCT with 52 TBI subjects suffering from major depressive disorder (MDD) by DSM-IV criteria [27]. Patients were started on sertraline 25 mg/day for one to two weeks followed by dose adjustment up to 100 mg/day. Medications were reassessed at two-week intervals and treatment responders were quantified by a 50% drop in Ham-D score, or a final Ham-D score of below 10. Treatment response rate was 59% compared to 32% for placebo. The authors noted a significant time effect for improvement of depression in both groups, and time as a confounder illustrates the inherent challenge in delineating the magnitude of SSRI treatment effect versus the natural course of mood recovery following TBI [27].

Lee and colleagues conducted a four-week double-blind parallel group trial with three treatment arms: placebo, sertraline (25–100 mg/day), and methylphenidate (2.5–20 mg/day) [30]. Thirty subjects with mild to moderate TBI injured between two weeks to one year previously who met DSM-IV criteria for MDD were randomized. Post-hoc analysis adjusting for group and time effects found that both sertraline and methylphenidate significantly improved depressive symptoms as assessed by the Ham-D and Beck Depression Index (BDI). Sertraline was associated with a higher rate of adverse events, and decreased performance on several cognitive tasks, compared to methylphenidate and placebo. The ambiguity of these results and the potential for side effects show that the treatment of depression and other behavioral outcomes with SSRIs in the TBI population continue to require large-scale RCTs to become generalizable [57].

An RCT focused on depression and quality of life (QoL) was conducted by Ansari and colleagues in 80 male patients with post-TBI depression randomized to sertraline or placebo, with sertraline showing significant improvement in depressive symptoms and QoL at three, six and nine months. Interestingly, patients suffering left-sided brain injury were more prone to developing post-TBI depression in this cohort [26].

Perino and colleagues conducted a prospective cohort study of 20 post-TBI patients with major depression diagnosed by two neuropsychiatrists from a group of 37 consecutive TBI patients undergoing counseling [40]. The patients were classified by time elapsed from trauma (Group A, <6 months; Group B, 24–36 months) and received combination therapy of carbamazepine 600 mg/day and sertraline 20 mg/day for 12 weeks. Carbamazepine is an anticonvulsant known to temper volatile mood. The brief psychiatric rating scale (BPRS) and the clinical global impression scale were recorded at baseline and after 12 weeks. Both the brief psychiatric rating scale and the clinical global impression outcomes showed statistically significant improvement in all patients, although Group B did not recover as well as Group A. Thought disorganization, depression, and psychomotor retardation showed the highest magnitude of improvement [40].

Fann and colleagues conducted a nine-week, nonrandomized single-blind trial of sertraline in 15 patients with MDD 3–24 months after mild TBI. Patients received placebo in week zero to eliminate “placebo responders”, and were dosed with 25–150 mg/day sertraline in the following eight weeks. Thirteen patients improved their overall Ham-D score by 50% and 10 demonstrated full remission with low/nonclinical Ham-D scores, indicating sertraline as an effective treatment of post-TBI depression [35,54].

Turner-Stokes and colleagues assessed response to sertraline in patients with severe disability following TBI in an integrated care rehabilitation facility. Patients that screened positive for depression using the BDI were treated for six to eight weeks. Sertraline significantly improved BDI scores even in patients who had previously been unresponsive to SSRI therapy [42].

#### 3.1.3. Meta-Analysis

**Figure 2:**
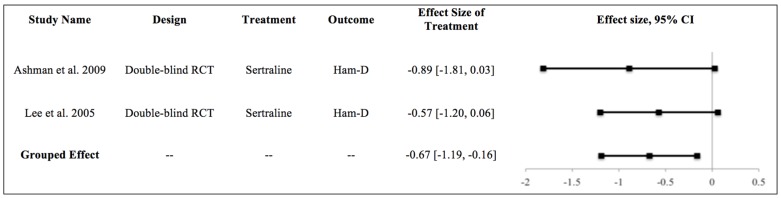
Meta-analysis of all double-blind, randomized controlled trials of sertraline versus placebo (*n =* 2), using the Hamilton Depression Rating Scale as the outcome. Effect sizes are reported for the treatment group (sertraline). CI, confidence interval; Ham-D, Hamilton Depression Rating Scale; RCT, randomized controlled trial.

Of the included studies in this manuscript, only two were RCTs utilizing the same intervention (the SSRI sertraline) (Ashman et al., 2009: 22 TBI subjects/19 controls; Lee et al., 2005: 10 TBI subjects/10 controls) and shared a common endpoint (Ham-D) to qualify for meta-analysis [27,30]. Using the random effects model, a statistically significant pooled effect size difference (Hedge’s *g*) of −0.67 (95% CI [−1.19, −0.16]; *p* = 0.011) was found for sertraline compared with placebo (Figure 2), demonstrating that sertraline administration following TBI was associated with decreased depression burden as assessed by Ham-D scores.

#### 3.1.4. Evidence Synthesis: Citalopram

One RCT and four open-label studies investigated the use of citalopram to treat depression. Rapoport and colleagues conducted an open-label study to investigate the efficacy of the SSRI citalopram in treating post-TBI depression in 65 patients with mild to moderate TBI, 54 of whom met DSM-IV criteria for MDD. The study had two arms: 29 subjects underwent a six-week trial receiving citalopram 20 mg/day, and 36 subjects underwent a six-week trial receiving citalopram on a flexible dosing schedule of 20–50 mg/day, which was extended to 10-weeks for a subset of individuals. Ham-D scores improved significantly at six-weeks across the overall cohort, and no statistical difference was found between scores at six vs. 10 weeks. The authors concluded that flexible dosing and increased treatment time improved Ham-D scores more than rigid dosing and/or short treatment time [41]. Twenty-one patients who met criteria for remission on the Ham-D following the 10-week treatment course were subsequently enrolled in a double-blind RCT where 11 patients were given citalopram and 10 were given placebo. Over 40 weeks of follow-up, no significant difference was noted between groups in time to relapse or relapse rates (~50%). The fact that nearly half of subjects in the study experienced relapse suggests increased susceptibility of TBI patients for depressive episodes [67,68]. There is a need to tailor treatment protocols to the time course of recovery and for early intervention, as response to SSRI has been shown to be most favorable during the subacute phase of recovery [33].

Lanctot and colleagues evaluated six serotonergic SNPs in 90 TBI with MDD diagnosed using the DSM-IV, including 5-HTT, 5-HT1A, 5-HT2A, TPH2, BDNF, and MTHR [69]. Patients received 20 mg/day of citalopram for six weeks and were assessed using Ham-D pre- and post-treatment. Patients with a Ham-D decrease of >50% were deemed treatment-responsive. Overall, patients demonstrated a mean decrease in Ham-D; the percentage of responders and remitters (23%, 17% respectively) were relatively small, indicating potentially suboptimal treatment doses. BDNF and MTHR polymorphisms significantly predicted improved treatment outcomes on Ham-D while 5-HTT polymorphisms predicted greater incidence of adverse effects to treatment [37].

Fleminger and colleagues describe in a review article numerous clinical factors for consideration in TBI longitudinal care, including the 40% incidence of depression and four-fold increase in suicide rates compared to the general population [55]. Specifically, a Chinese study highlights the utility of managed care in 68 patients with clinically diagnosed depression following TBI. After initial psychotherapy, eight subjects demonstrated remission; remaining subjects were treated with citalopram alone (*n =* 28), or citalopram plus prednisone combination due to hypocortisolism (*n =* 32) [38]. Both groups demonstrated remission rates >50%. This adds to the evidence base that future studies targeting combination therapy for individuals suffering post-TBI depression should certainly include SSRIs.

#### 3.1.5. Evidence Synthesis: Fluoxetine

Fluoxetine has been shown to stimulate BDNF expression and potentiate neuronal remodeling and elongation of CA3 pyramidal neurons in rodent hippocampi, which may lead to improved memory and executive function. One case report investigated the use of fluoxetine to treat depression. Horsfield and colleagues investigated five TBI patients with variable severities and depressive symptoms who were provided fluoxetine on a flexible dosing schedule (20–60 mg/day) for eight months. The authors found significant improvement on the Ham-D and tests of executive function and mental flexibility compared to pre-treatment [36].

#### 3.1.6. Commentary

Indications for SSRI to treat depression have been well-studied with a robust evidence base, and not surprisingly finds concurrence in our review. The narrative of an acute therapeutic window for SSRI administration in TBI is supported by multiple RCTs and cohort studies [26,27,29,41,54,56,66], with an estimated reduction ~50% in symptom severity and/or remission rate over the first two to three months of SSRI administration. It would appear that, at least preliminarily such improvements are sustained [26,33,54]. There is preliminary evidence suggesting benefit in prophylactic administration of SSRI in TBI patients without baseline depression, foremost from the RCT by Jorge et al. with a NNT of 5.9 persons treated for one depression case prevented at 24-weeks post injury [29]. However across the majority of studies, relatively smaller sample sizes, low rates of depression as compared to the overall TBI population, and inherent challenges in drawing conclusions based on prophylactic studies [32] highlight the need for larger RCTs studying the ideal time course for SSRI administration post-TBI in patients with and without baseline mood disorders.

The inability to clearly separate treatment effect from the natural time course of TBI recovery is a significant limitation in multiple trials. To this end, a placebo-controlled imaging study examining functional and/or neurochemical changes during the treatment course can assist in identifying modulatory changes associated with SSRI [36]. Sample size remains another key issue—although individually the studies by Lee et al. and Ashman et al. showed no treatment effect for sertraline versus placebo, when the studies were pooled using a random effects model, a significant pooled effect size difference of −0.67 was demonstrated between sertraline and placebo. While the limitations of this meta-analysis include small sample sizes and differing time to follow-up, for the first time there is pooled preliminary evidence for the benefit of sertraline to treat post-TBI depression.

### 3.2. Cognition

#### 3.2.1. Background and Pathophysiology

Following mild TBI, memory and information processing faculties are most vulnerable while language and intellect are relatively well preserved. On average, cognitive deficits resolve one to three months following mild TBI but can persist in moderate to severe TBI patients for 12 months or more [70]. While the relationship between TBI, chronic traumatic encephalopathy and dementia remain in need of further characterization, a number of studies now show that latent cognitive deficits can surface years after the initial insult [71]. Sixty-three percent of moderate to severe TBI patients report long-term cognitive impairment and nearly a quarter are unable to return to the workforce. Although some cognitive deficits can be localized neuroanatomically, the sources of others remain poorly understood in part due to confounding from behavioral traits.

Cognitive performance is generally measured by task performance on scales e.g., working memory, verbal fluency, reasoning, judgment, visual construction, and verbal recall [72]. Higher cognitive functioning is associated with improved life satisfaction, QoL and functional independence [73,74]. The unfavorable cognitive side effect profile of other antidepressant class drugs, such as TCAs and monoamine oxidase inhibitors (MAOIs), have propelled SSRIs to the forefront of therapeutic options [59]. Thus, fully understanding the impact of SSRIs on recovery and cognition is critical.

#### 3.2.2. Evidence Synthesis

Ongoing ambiguity exists regarding the impact of SSRIs on cognitive recovery. Three RCTs and one open-label study investigated the use of sertraline, and one open-label study investigated the use of fluoxetine, to improve post-TBI cognition. In an RCT by Meythaler and colleagues, 11 severe TBI patients received sertraline 100 mg/day vs. placebo for two weeks after injury and showed no significant differences on measures of attention and orientation [31]. In another double-blind RCT, Baños, Novack and colleagues examined cognitive recovery with early sertraline administration in 99 patients (sertraline *n =* 49, placebo *n =* 50) for three months following TBI. No differences were observed between the groups on measures of cognitive and executive function as assessed by the Neurobehavioral Functioning Inventory (NFI) and other standard measures at three, six and 12 months post injury [28,32]. As previously described, Lee and colleagues compared the effects of sertraline, methylphenidate and placebo on mild-to-moderate TBI patients in a four week double-blind RCT and demonstrated that the sertraline group performed worse on a number of cognitive tests including the Mini-Mental Status Exam (MMSE), while the methylphenidate group scored better than placebo [30]. In a recent meta-analysis, Wheaton and colleagues describe reductions in psychomotor speed, reaction time and memory attributed to sertraline administration during postacute recovery from TBI [61]. Interestingly, evidence in cohort studies for SSRI effects has been generally positive. Fann and colleagues conducted an eight week non-randomized, single-blinded trial for sertraline 3–24 months following mild to moderate TBI, in patients meeting DSM-IIIR criteria for MDD. Thirty-two patients were evaluated with a neuropsychiatric battery and across 24 tests, 14 showed post-treatment improvement including digit symbol, finger-tapping, executive function, memory recall, visual retention, and self-perception of injury severity. Of importance, the placebo run-in design of this study mitigates the confounding time effect for natural recovery of cognitive symptoms [75]. A smaller study by Horsfield and colleagues in five TBI patients reported that fluoxetine improved performance on tests of executive function, attention, and nonverbal intelligence [36].

#### 3.2.3. Commentary

The evidence to date for SSRI effects on cognition is equivocal to detrimental in RCTs, while cautiously beneficial in select measures of nonverbal cognition in cohort studies. The evidence to date for SSRI effects on cognition is equivocal to detrimental in RCTs, while cautiously beneficial in select measures of nonverbal cognition in cohort studies. Murine models have demonstrated that the neurobiological effects of SSRIs are mediated via hippocampal neurogenesis, whereas irradiation of the hippocampus-containing region prevents the influence of antidepressants [76,77,78]. Chronic treatment using paroxetine appears to improve temporal order memory performance compared to controls [79]. In patients with depression, fluoxetine has been shown to improve memory function without altering hippocampal volume [80].

The effect of SSRI administration on depression and cognition—two areas of great importance to the TBI population—are similarly ambiguous. In considering study design, the included studies lack the adequate time of follow-up necessary to separate drug effects from the natural course of cerebral and mental health recovery. Although a few studies showed improvement in psychomotor speed and various attention/memory tasks, no study with a placebo control demonstrated cognitive improvement attributable to SSRI use. Another consistent limitation is the inability to measure one’s cognitive function and/or reserve prior to suffering TBI. Notably, the three-group, parallel design study by Lee et al. demonstrated that while SSRIs led to improvement in depressive symptoms over the course of the trial, the aggregate cognitive function as assessed by numerous tests was significantly lower than patients without SSRI treatment. This finding raises important considerations for the administration of SSRIs post-TBI, as medical professionals struggle to balance the alleviation of depressive symptoms with the possible worsening of cognitive ability/processing speed. This tradeoff suggests that SSRIs may benefit specific subpopulations of patients while being a detriment to others.

### 3.3. Post-Concussive Symptoms/Syndrome

#### 3.3.1. Background and Pathophysiology

Mild TBI comprises 70%–80% of TBI. The constellation of symptoms following TBI, including headaches, dizziness, irritability, fatigue, depression, and others, are diagnostically and colloquially termed “post-concussive symptoms”, and if debilitating/disruptive enough to functional, social and/or cognitive activity, “post-concussive syndrome (PCS)” [81,82,83]. No FDA-approved treatment currently exists for PCS [84,85], and options for treating specific symptoms may or may not be beneficial for treating PCS as a whole. As SSRIs are the first-line treatment for depression—a component of PCS—it is of interest to assess whether they are indicated in subpopulations of PCS sufferers.

The current standard of measurement for PCS is the Rivermead Post-Concussional Symptoms Questionnaire (RPQ), assessed three months after TBI [86]. The RPQ consists of 16 self-report symptoms and a score of “moderate severity” or above in any three symptoms constitutes PCS as assessed by the DSM-IV [86,87], which has been well validated in mild TBI patients [88,89,90].

#### 3.3.2. Evidence Synthesis

One RCT investigated the use of sertraline to treat PCS, and one open-label study investigated the use of citalopram.

Lee and colleagues conducted a four-week, three-arm RCT (sertraline, methylphenidate, placebo) in 30 adults following mild-to-moderate TBI and found improved post-study RPQ scores in both methylphenidate and placebo groups but not in the sertraline group compared to baseline. One confounder is that the mean baseline RPQ score for the sertraline group was lower than the other two groups [30].

In a prospective cohort study of 54 patients suffering a major depressive episode or MDD following mild-to-moderate TBI, Rapoport et al. found a significant mean decrease in RPQ scores after six weeks of treatment with citalopram 20–50 mg/day, with a treatment response rate of 28% at six weeks and 42% at 10 weeks. There was no change RPQ score at the end of 10 weeks of treatment compared to six weeks, suggesting that while citalopram effect persisted, the attenuation of PCS symptomatology was highest during the subacute phase of recovery [41]. The study shows the promise for SSRIs as treatment for components of post-concussive symptomatology, however remains limited by the lack of placebo and of control for concurrent psychotropic medications [41].

#### 3.3.3. Commentary

To date, current data regarding the use of SSRIs to treat PCS are minimal and limited. Part of the issue stems from the definition of PCS which is heterogeneous consisting of somatic, depressive, anxiety, and cognitive components. As evidenced in our review, SSRIs have been shown to improve select symptom clusters, e.g., depression, but remain equivocal to detrimental for others, e.g., cognition. Hence, further research is needed to identify the subpopulation of PCS patients that would benefit from SSRI treatment and assess its comparative efficacy and tolerability at different phases of recovery. In considering combination therapy, it is posited that serotonin inhibits dopaminergic neuronal projections in the midbrain and forebrain while methylphenidate promotes release and prevents reuptake of dopamine. Consequently, SSRIs and methylphenidate may have opposing effects in sleep induction and/or preservation in the context of PCS [91].

As with all TBI studies, heterogeneous cerebral pathophysiologies and severities have hindered the practical design of large-scale RCTs [58]. Clinicians are often subjected to difficult treatment decisions, as the small sample sizes and uncontrolled investigation designs of published studies leaves TBI treatment to clinical practice and experience rather than evidence-based protocol [60]. In considering a complex, multifactorial clinical syndrome such as PCS, first steps toward proper assessment of SSRI utility and efficacy should be reserved for well-designed, controlled trials targeting similar TBI subtypes rather than encompassing the spectrum of TBI [57].

### 3.4. Sleep Disturbance

#### 3.4.1. Background and Pathophysiology

It is estimated that sleep disturbances impact 30%–70% of patients following TBI [92] with drastic implications on mood and emotional regulation. Sleep disorders often arise from secondary complications after TBI, and can manifest via sleep apnea, hypersomnia, circadian rhythm disorder, PTSD, depression, anxiety and pain. The type of sleep disturbance is often associated with the location of axonal shearing in the brain; damage to the ventrolateral preoptic nucleus, suprachiasmatic nucleus, basal forebrain and posterior hypothalamus has been associated with sleep disorders in TBI patients [93]. Correction of sleep disturbance is a critical component of symptom management for TBI patients [27].

Biochemically serotonin is a precursor to melatonin, a neurohormone with paracrine activity in the suprachiasmatic nucleus of the hypothalamus to regulate sleep/wake cycles [94]. The biological conversion of serotonin into melatonin for induction of sleep highlights the potential for serotonin turnover to alter sleep cycles.

The exact role of serotonin in sleep regulation is controversial. Serotonin levels are elevated in most cortical areas during wakefulness and accordingly, SSRIs have been shown to cause insomnia, delay onset of rapid eye movement (REM) sleep, increase nighttime awakenings and reduce both REM and slow-wave sleep [95]. SSRI-induced insomnia may be related to stimulation of the 5-HT2 receptors in the brain sleep centers, part of the serotonergic pathway that projects to the cholinergic neurons of the lateral tegmentum, which are critical for inhibition and sleep maintenance [96,97]. High serotonin levels during wakefulness and high activity in serotonergic neurons of the dorsal raphe nucleus further contribute to serotonergic control of waking cortical activity [97].

#### 3.4.2. Evidence Synthesis

Few studies have been conducted specifically to study SSRI effects on sleep following TBI. The aforementioned three-group (sertraline, methylphenidate, placebo) RCT by Lee et al. found methylphenidate to be better at reducing daytime sleepiness whereas sertraline provided no benefit over placebo [30].

#### 3.4.3. Commentary

It is logical that a nootropic stimulant can improve alertness; methylphenidate works by promoting the release of dopamine from presynaptic terminals and preventing its reuptake by presynaptic terminals [98]. The increased concentration of extraneuronal dopamine impacts the basal ganglia and cortex promoting alertness and wakefulness. Of note, Sobanski et al. found that in adults with sleep disturbance due to ADHD, methylphenidate improved sleep quality as well [99].

The complex mechanism of serotonin involvement in regulation of sleep underlies the diverse side effects attributable to SSRI treatment. The utility of SSRIs as part of combination therapy to treat post-TBI depressive symptoms while avoiding insomnia and sleep-cycle disruptions remains an active area of investigation. Future well designed controlled cohort studies or RCTs of such therapies are necessary to understand better drug interactions and risk profiles for different age groups, comorbidities, professional responsibilities and support networks, and emotional resiliency in the TBI population.

### 3.5. Anxiety Spectrum Disorders

#### 3.5.1. Background and Pathophysiology

Anxiety symptoms following TBI may be due to a combination of neurotransmitter disruption and neuronal dysregulation, psychological factors associated with trauma, and social consequences from TBI. Symptoms range from subtle changes in mood and behavior to debilitating panic, agitation, and agoraphobia, often most profound shortly following injury. As expected, preexisting psychiatric conditions increase the risk of developing new anxiety disorders following injury [100,101]. At one-year post injury for mild TBI, the new-onset anxiety disorders have been reported as follows: general anxiety disorder 10%, PTSD 7.8%, agoraphobia 7.2%, social phobia 4.9%, panic 3.7%, and OCD 1.8% [102].

#### 3.5.2. Evidence Synthesis

One open-label study and seven case reports investigated the use of SSRIs in post-TBI anxiety spectrum disorders, including obsessive-compulsive disorder (OCD), panic disorder, and emotional incontinence.

#### 3.5.3. Obsessive-Compulsive Disorder (OCD)

SSRIs are the first-line treatment for OCD due to documented advantages over placebo [103,104]. Across three studies with 282 patients and a maximum follow-up period of 7.5 years, TBI was associated with a 2.6-fold increase in relative risk of developing OCD [105]. In a 2002 case report Stengler-Wenzke and colleagues reported on an 18-year-old man who sustained a TBI due to a motor vehicle accident (MVA). Ten months after the initial trauma, the patient recovered normal neurological function but reported compulsions, obsessions and impulsivity, scoring far above the threshold for clinical screening on the Yale-Brown Obsessive Compulsive Scale (YBOCS). His MRI showed multiple lesions affecting the right ventrolateral prefrontal cortex, and single-photon emission computed tomography (SPECT) imaging showed serotonin transporter density at two SDs below normal. After nine days of treatment with 60 mg of fluoxetine/day the patient reported marked reduction of compulsions with decreased YBOCS [51]. The fact that these studies suggest a similar role of SSRIs for TBI-induced OCD raises the need for an RCT or at minimum a large controlled cohort study to evaluate their efficacy in modulating post-TBI OCD development.

#### 3.5.4. Panic Disorder

In the three aforementioned studies by van Reekum and colleagues, TBI was associated with a 5.8-fold relative risk for developing panic disorder [105]. Similarly, a 2010 prospective cohort study of 1084 individuals showed two-fold odds of developing panic disorder after TBI [102]. Damage to the frontal lobes, in particular the ventral medial prefrontal cortex, which modulates fear response in the amygdala, and/or direct compromise to the hippocampus and amygdala, are implicated in the development of post-TBI panic and anxiety disorders [102]. In a case report Scheutzow et al. described an individual suffering a panic attack during rehabilitation three months following TBI, which negatively impacted lifestyle, employment, relationships, and increased risk of suicide. While sertraline administration for two months improved mood and appetite symptoms, it did not lead to full resolution of panic and anxiety symptomatology; in these refractory cases cognitive behavioral therapy may be additionally indicated [46].

#### 3.5.5. Emotional Incontinence

The true prevalence of emotional lability and/or incontinence following TBI is difficult to discern due to high rates of concurrent neuropsychiatric sequelae e.g., depression and/or mania. Nahas et al. report a case of emotional incontinence in a 21-year-old Caucasian man sustaining severe TBI after motor vehicle ejection; at six months he continued to suffer from memory, impulse control and language deficits as well as outbursts of inappropriate hysterical laughter; notably, administration of fluoxetine 20 mg/day decreased symptomatology in two days and resolved all symptoms within one week [44]. In another 56-year-old female TBI patient requiring bilateral frontal lobectomies who exhibited labile irritability and spontaneous crying refractory to lithium carbonate, administration of sertraline 50 mg/day completely resolved her symptoms of emotional lability and depression [52]. Additionally, two cases of post-TBI Kluver-Bucy syndrome—a mood disorder characterized by hypersexuality and hyperorality due to medial temporal lobe and amygdala lesions—were successfully treated with SSRI [47].

#### 3.5.6. Commentary

In persons with TBI presenting with anxiety symptoms, pharmacotherapy using sertraline may serve as effective adjuncts to behavioral modification and cognitive-behavioral therapy in treating specific anxiety disorders, although few studies have been able to accurately measure the efficacy following TBI due to frequent co-occurrence with depression [27,35]. Studies within the scope of this review consist of case reports supporting the efficacy of SSRI in reducing anxiety following TBI, hence results from post-TBI SSRI therapy are individual-based and dependent on the presenting symptoms.

### 3.6. Side Effects of SSRI Administration

#### 3.6.1. Depression-Indicated Studies

The need for a comprehensive system of care is demonstrated by numerous case reports outlining side effects associated with SSRIs in post-TBI depression. Patterson and colleagues describe a 43-year-old man treated with fluoxetine following moderate TBI, who developed dysarthria and speech block within one week as well as insomnia and needed supplemental trazodone [45]. Similarly, a 24-year-old man receiving sertraline for PTSD reported dystonia who was also administered trazodone [50]. Spinella et al. describe a 42-year-old woman with a history of depression following mild TBI who combined St. John’s Wort with buspirone and fluoxetine, leading to hypomania characterized by insomnia, worsening memory, agitation and increasing depression [49]. Side effects in all cases ceased upon discontinuing SSRI, underscoring the need for caution and mindful evaluation when prescribing SSRIs with other psychotropic medications. In contrast, a 23-year-old TBI patient who developed seizures after the TCA desipramine experienced resolution when switched to fluoxetine and phenytoin [53]. A study of 10 mild to moderate TBI patients with depression found that Ham-D and MMSE scores significantly improved after six weeks of treatment with the serotonin noradrenaline reuptake inhibitor (SNRI) milnacipran, which does not inhibit CYP450 subtypes and therefore cause fewer drug interactions compared to SSRIs [106].

#### 3.6.2. Akathisia

Akathisia is defined as a feeling of inner restlessness resulting in the inability to remain still, and the compulsion to move with observable restless movements. Hensley and colleagues describe the case of a 22-year-old male with history of polysubstance abuse and PTSD who sustained TBI with polytrauma after MVA [43]. The patient was started on sertraline 50 mg/day for anxiety and depression and demonstrated extreme restlessness and anxiety two days into administration. Similar restless symptoms occurred with administration of the SSRI paroxetine, further raising suspicion for akathisia as an early adverse effect of serotonergic toxicity. Ultimately, a TCA was prescribed and restlessness did not recur [43]. It is hypothesized that serotonergic toxicity occurred in this case due to serotonergic inhibition of dopamine pathways. The mechanism of akathisia is not well understood and may depend on drug metabolism through CYP450, serotonin receptor affinity and the selectivity of the administered SSRI [107].

#### 3.6.3. Sexual Dysfunction

A common barrier to treatment using SSRIs is the associated side effect of sexual dysfunction. Mechanistically, serotonergic inhibition of excitatory mesolimbic dopaminergic pathways, CYP450-mediated decreases in nitric oxide, and anticholinergic effects correlate with a 30%–50% prevalence of sexual difficulties in patients using SSRI [108]. Dolberg and colleagues report that 15 of 17 TBI patients prescribed SSRIs showed improvement in sexual dysfunction with concurrent administration of the 5-HT2A postsynaptic agonist mianserin, 10 of whom reached pre-injury levels of sexual function [34]. Although isolated, this study underscores an important area of future research regarding the potential for combination therapies utilizing the benefits of SSRIs following TBI if the side effects are well controlled.

### 3.7. Post-Traumatic Stress Disorder (PTSD): Commentary and Implications

PTSD is a clinical syndrome characterized by reliving trauma, self-isolation and avoidance, and hypervigilance in response to severe traumatic events. Clinically significant changes in arousal, cognition and mood converge with the overlapping neuropsychiatric sequelae with mild and moderate TBI [109]. PTSD is often associated with functional changes in the amygdala, hippocampus, orbitofrontal, dorsomedial and dorsolateral prefrontal cortex [110]. Of these areas, the orbitofrontal, dorsomedial, dorsolateral prefrontal cortex and the hippocampus are prone to axonal shearing following TBI. Common neurobiological etiology supports an extensive overlap of symptoms including sleep disturbance, cognitive impairment, depression and anxiety between TBI and PTSD [111].

The FDA-recommended treatment for PTSD includes the SSRIs sertraline and paroxetine based on RCT evidence [112,113]. Fluoxetine, venlafaxine (SNRI, serotonin and norepinephrine reuptake inhibitor) and prazosin (alpha-1 adrenergic receptor antagonist) have also shown promise in treating PTSD [109,114,115]. For this reason, SSRI treatment of comorbid TBI and PTSD is important to guide physician care of the growing number of patients presenting with this complex constellation of symptoms.

Of note, our review did not identify any RCTs or prospective studies targeting the efficacy and utility of SSRI administration to treat symptoms specifically following TBI. While there is ample evidence for SSRI use in treatment of PTSD, the lack of evidence-based trials for SSRI use in the post-TBI population is troubling and should be a pressing focus of further studies. From the RCTs discussed in our review, the threshold for conducting such a study in this population is reasonable and within reach.

## 4. Discussion

The rising incidence of TBI, coupled with the increased survival after TBI (due to improvements in medical management), creates a pressing need for further study into the best practices for managing the psychiatric sequelae of TBI. This comprehensive review of the literature to date review investigated the utility of SSRIs in treating a constellation of post-TBI cognitive, psychiatric and mental health disorders. SSRIs are widely prescribed due to their efficacy, relatively selective mechanism of action, and lower side effect profile compared to earlier antidepressants (e.g., monoamine oxidase inhibitors). We aimed to consolidate the literature describing the effects of SSRI following TBI. It is our hope that, by summarizing the state of the current knowledge base, we highlight the need for further research toward generating guidelines for SSRI use specifically for treatment of TBI sequelae. Our goal is to ensure that SSRIs are utilized and prescribed in the correct manner in order to optimize recovery following TBI.

### 4.1. Limitations

This comprehensive review on the management of neuropsychiatric disorders following TBI using SSRIs has demonstrated several important limitations. Due to the breadth of studies available, we present our findings as an exploratory investigation without grading the level of evidence or bias, which is a limitation of the current study. We also perform a small meta-analysis of disorders sharing a common medication and a common endpoint, and in our literature search this was limited to sertraline and the Ham-D, respectively. The small sample sizes of the RCTs and the open-label trials have hampered the ability to provide definitive efficacy of SSRI in treating neuropsychiatric deficits other than post-TBI depression. There is a lack of retrospective cohort studies on the topic, which may bolster Class III evidence. Notably, in the current report we did not evaluate the effect of SSRIs in the setting of and/or compared to other interventions such as cognitive behavioral therapy, which warrants further study. These constitute relevant topics for future investigations in the manner of a systematic review, which can be performed for specific medications, disorders and/or outcomes using a more targeted approach and/or in greater depth.

Other pharmacotherapies including methylphenidate, dextroamphetamine, amantadine, and tricyclic antidepressants are also being studied as part of pharmaco-rehabilitation for the improvement of cognition, mood, and behavior in various TBI subpopulations [116,117,118]. Further, Van Waes and colleagues highlighted through the expression of transcription factors that SSRIs might not only influence depression and cognition, but also enhance the addition potential for methylphenidate when used in combination [119]. Variations in time to follow-up after initial trauma, in addition to the inability to clearly delineate treatment effects from the natural course of recovery, are also significant limitations that can be overcome through using consistent outcome measures, standard follow-up intervals, larger sample sizes, and systematic blinding of and/or control of cohorts to generate Class I and II evidence. Lastly, the lack of a cognitive baseline prior to injury is a known limitation of the accurate assessment of cognitive deficits attributable to trauma.

### 4.2. Future Directions

While SSRIs are indicated as first-line treatment for a variety of psychiatric disorders both in general and following TBI, the small effect sizes associated with SSRI treatment of post-TBI depressive symptoms in a placebo-controlled setting demonstrates the need for further research, with standardized patient populations and larger sample sizes. Potential deleterious effects of SSRIs on cognition and agitation when used in conjunction with other psychotropic agents, along with its known side effects and interactions with medications processed by CYP450, demonstrate the need for careful cost-benefit assessment of SSRI use following TBI. Therefore, combined with the lack of current consensus for improved outcomes, SSRIs remain a “potential option” rather than “effective treatment” in the TBI population.

The heterogeneity of TBI, coupled with the broad effect of SSRIs on brain structure and function, may require more selectively controlled populations and sophisticated trial methodologies in future studies. We recommend a placebo-controlled longitudinal study with an extended follow-up period at multiple time points, along with concurrent biomarker, neuroimaging and behavioral data to delineate the true pharmacological effect of SSRIs in the TBI population. Absent such a study, SSRIs must be applied on a case-by-case basis after careful attention is paid to the specific mood, behavioral, and cognitive complaints of a given patient.

## 5. Conclusions

In the TBI population, RCTs on the efficacy of SSRIs have been few and limited to the specific outcome measures of depression and cognition. SSRI use is generally associated with improvements in depressive symptoms, although this effect may be a result of natural cerebral recovery. Conversely, SSRI administration was not found to have any benefit on cognition; in fact, it may worsen cognitive function. Pooled results using the Ham-D and sertraline showed a statistically significant mean decrease in depressive symptoms as compared to placebo. SSRIs are broadly utilized in a number of case reports and small open-label studies after TBI with general improvements in mood symptomatology, and absent or negative effects for cognition and functional recovery. There is a dearth of studies focusing on SSRI treatment effects for PTSD after TBI. Given the status of SSRI as a first-line therapy for general PTSD, discernment of SSRI efficacy for TBI-induced PTSD represents an important direction of future research. Parallel studies for other neurocognitive and neuropsychiatric domains are equally important in assessing the potential of SSRIs to reduce mental health burden and optimize recovery across TBI subpopulations.

## Figures and Tables

**Table 1:** Summary of studies.

**Randomized-Controlled Trials**						
**Author**	**Treatment**	**Description**	***N***	**Endpoints**	**Results**	**Post-TBI Disorder**
Ansari et al., 2014 [26]	Sertraline	80 adult male patients with post-TBI depression. 40 patients given sertraline 50 mg/day, 40 patients given placebo.	80	PHQ-9	Sertraline group showed significant improvement in mood and QOL domains (PHQ-9: 14.88 ± 3.60 vs. 5.33 ± 2.98, *p =* 0.040).	Depression
Ashman et al., 2009 [27]	Sertraline	10-week program studying 52 patients with TBI and MDD treated with sertraline or placebo.	52	Ham-D	Both groups significantly improved (59% treatment group, 32% placebo group) with Ham-D reduction by 50%.	Depression
Banos et al., 2010 [28]	Sertraline	Three-month study of 99 subjects with moderate/severe TBI randomized to sertraline 50 mg (*n =* 49) or placebo (*n =* 50).	99	WMS, TMT, NFI	No sertraline treatment effect was observed for cognitive performance.	Cognition
Jorge et al., 2016 [29]	Sertraline	94 patients administered sertraline vs. placebo at 100 mg/day for 24-weeks.	94	MINI	Number needed to treat to prevent depression after TBI at 24-weeks is 5.9 for sertraline vs. placebo (*p =* 0.03). Sertraline effects were well-tolerated.	Depression
Lee et al., 2005 [30]	Sertraline	Four-week study of 30 patients with MDD treated with sertraline 25–100 mg/day (*n =* 10), methylphenidate 5–20 mg/day (*n =* 10), or placebo (*n =* 10).	30	Ham-D, ESS, RPQ	Methylphenidate and sertraline showed improvement in depressive symptomatology. Methylphenidate and placebo showed improvement in cognitive function vs. sertraline.	Cognition, Depression, PCS
Meythaler et al., 2001 [31]	Sertraline	Two-week study of 11 patients with severe TBI post MVA. Patients received sertraline 100 mg/day or placebo.	11	OL, ABS, GOAT	No effect of sertraline treatment was identified.	Cognition
Novack et al., 2009 [32]	Sertraline	One-year study of 99 non-depressed TBI subjects received either sertraline 50 mg/day (*n =* 49) or placebo (*n =* 50).	99	Ham-D, NFI	Placebo group developed more depressive symptoms (*p =* 0.023). Sertraline associated with decreased neurobehavioral functioning.	Depression
Rapoport et al., 2010 [33]	Citalopram	21 patients in remission from depression after TBI were randomized to same-dose citalopram (*n =* 10) or placebo (*n =* 11) and followed for 40 weeks.	21	Ham-D	Relapse occurred in 11 subjects (52.4%). Treatment groups did not differ in relapse rates (citalopram: 50% vs. placebo: 54.5%; *p =* 0.835). This trial suggested limitations of pharmacotherapy in the prevention of MDD relapse following TBI.	Depression
**Open-Label Studies**						
**Author**	**Treatment**	**Description**	***N***	**Endpoints**	**Results**	**Post-TBI Disorder**
Dolberg et al., 2002 [34]	Fluoxetine, Citalopram, Paroxetine, Sertraline	17 TBI patients were given SSRIs. All complained of sexual dysfunction which was resolved with mianserin (tetracyclic anti-depressant).	17	Occurrence of sexual dysfunction	SSRI use associated with sexual dysfunction. 15 patients (88%) reported improvement of symptoms with mianserin.	Sexual dysfunction
Fann et al., 2000 [35]	Sertraline	15 patients with mild TBI within the past 3–24 months. Placebo in-run design where all subjects received 1-week placebo followed by 8-week single-blind course of sertraline.	15	Ham-D	Sertraline significantly improved depressive symptoms (Ham-D 25.0 ± 4.4 to 7.2 ± 5.3 at Week 8 (*p* < 0.001). There was improvement of cognitive functions in psychomotor speed, cognitive efficiency, flexible thinking, and recent memory ability.	Cognition, Depression
Horsfield et al., 2002 [36]	Fluoxetine	5 TBI patients with no to moderate depressive symptoms followed for 8 months.	5	TMT, AMT, WAIS-III, USCREMT, MMSE, Ham-D	Fluoxetine improved mood and performance on some but not all cognitive measures. More studies needed.	Cognition
Lanctot et al., 2010 [37]	Citalopram	90 patients with major depressive episode following TBI in a six week study also examining six serotonergic SNPs.	90	Ham-D	MTHFR and BDNF SNPs predicted greater treatment response (r^2^ = 0.098, F = 4.65, *p =* 0.013). The 5HTTLPR SNP predicted greater occurrence of adverse events (r^2^ = 0.069, F = 5.72, *p =* 0.020). Serotonergic SNPs may associate with tolerability and efficacy of SSRIs.	Depression
Luo et al., 2015 [38]	Citalopram, prednisone	68 patients with depression following TBI.	68	Glasgow Coma Scale, Ham-D	Over 60% of patients who did not respond to psychotherapy alone (60/68) responded to citalopram treatment. Patients with hypocortisolism also were treated with prednisone	Depression
Muller et al., 1999 [39]	Paroxetine, Citalopram	26 patients with brain damage and pathological crying. Only 2 TBI related.	2	Clinical interviews related to pathological crying	Both paroxetine and citalopram improved symptoms for 24/26 (92.3%) patients within 3 days.	Emotional incontinence
Perino et al., 2001 [40]	Citalopram, Carbamazepine	20 patients with MDD following TBI were divided into two groups: group A with recent TBI (<6 months), and group B with long-term TBI (24–36 months).	20	BPRS	BPRS and CGI scores of the total sample showed significant improvement between baseline and 12 weeks (BPRS baseline: 62.3 ± 17.6 vs. 12 weeks: 51.7 ± 12.8; *p* < 0.05), (CGI severity-scale baseline: 4.4 ± 1.1 vs. 12 weeks: 3.4 ± 0.8; *p* < 0.005). No group effects were observed.	Depression
Rapoport et al., 2008 [41]	Citalopram	54 patients with mild to moderate depression post-TBI. 29 patients underwent 6 week fixed dose treatment; 36 underwent 10-week flexible dose treatment.	54	Ham-D	The mean Ham-D at baseline and 6 weeks were 23.66 (SD 6.8) and 16.30 (SD 9.3), respectively (*p* < 0.0001). The mean Ham-D at 10 weeks was 12.96 (SD 7.9) (*p* < 0.0001). Treatment showed significant reduction in depressive symptoms.	Depression, PCS
Turner-Stokes et al., 2002 [42]	Sertraline	27 patients with depression due to brain injury - 5 due to TBI.	27	BDI-II	The BDI-II was assessable in 17/21 patients, showing a mean improvement of 14.5 ± 9.7 (*p* < 0.001).	Depression
**Case Reports**						
**Author**	**Treatment**	**Description**	***N***	**Endpoints**	**Results**	**Post-TBI Disorder**
Hensley et al., 2010 [43]	Sertraline, Paroxetine	22-year-old female with MVA-related TBI.	1	--	Treatment of alcohol withdrawal related anxiety with SSRIs led to akathisia that resolved with TCA treatment.	Akathisia, Anxiety
Nahas et al., 1998 [44]	Fluoxetine	21-year-old male with MVA-related TBI leading to pathological crying.	1	--	Fluoxetine treatment led to complete resolution of pathological crying within 1 week.	Emotional incontinence
Patterson et al., 1997 [45]	Sertraline, Trazodone	43-year-old male with TBI following fall prescribed trazodone for chronic pain and sleep disturbance, and fluoxetine for treatment of depression.	1	--	Fluoxetine addition led to dysarthria that resolved with fluoxetine discontinuation.	Depression
Scheutzow et al., 1999 [46]	Sertraline	60-year-old male with TBI following MVA experiencing panic attacks.	1	--	Sertraline improved mood and appetite but did not resolve all panic and anxiety symptoms.	Anxiety, Panic
Slaughter et al., 1999 [47]	Sertraline	2 male patients with Kluver-Bucy syndrome from MVA related TBI.	2	--	Resolution of symptoms (hyperorality, hyper-sexuality) with high dose SSRI similar to OCD.	Emotional incontinence, OCD
Sloan et al., 1992 [48]	Fluoxetine	28-year-old assault victim with TBI related pathological laughter, dysarthria and hemiataxia.	1	--	Fluoxetine plus speech therapy helped with emotional lability grading and was well tolerated.	Emotional incontinence
Spinella et al., 2002 [49]	Fluoxetine, Buspirone, Ginkgo Biloba	42-year-old female with mild TBI following MVA.	1	--	Herbal supplements plus SSRI led to hypomania, highlighting the need to study SSRI interactions with other medications following TBI.	Depression
Stanislav et al., 1999 [50]	Sertraline	24-year-old with severe TBI following MVA presenting with PTSD.	1	--	Clinical case of dystonia following SSRI treatment.	Depression, PTSD
Stengler-Wenzke et al., 2002 [51]	Fluoxetine	18-year-old male with severe TBI following MVA.	1	--	Fluoxetine treatment drastically reduced OCD symptoms and increased quality of life.	OCD
Workman et al., 1992 [52]	Sertraline, Lithium	56-year-old female with severe TBI from fall requiring bilateral frontal lobectomies.	1	--	Sertraline plus lithium treatment reduced patients Ham-D score from 15 to 4. Symptoms of mood lability and conceptual disorganization resolved.	Emotional incontinence
Wroblewski et al., 1992 [53]	Fluoxetine, Phenytoin	23-year-old TBI patient with seizures following TCA treatment of depression.	1	--	Resolution of seizures and improvement of mood with fluoxetine treatment with phenytoin.	Depression
**Review Articles**						
**Author**	**Treatment**	**Description**	***N***	**Endpoints**	**Results**	**Post-TBI Disorder**
Fann et al., 2009 [54]	Review	--	--	--	Serotonergic system modulation through antidepressants has high tolerability in treatment of TBI patients with depression.	General
Fleminger et al., 2003 [55]	Review	--	--	--	Three- to four-fold increase in suicide rates following TBI, suggested heightened surveillance for TBI subpopulations. Little conclusive evidence for SSRI use highlights need for longitudinal care.	Depression
Jorge et al., 2003 [56]	Review	--	--	--	Mood disorders are frequent complications of TBI and are often overlooked. Further research is needed for the neuropsychiatric sequelae of these disorders.	Anxiety, OCD, Panic
Lee et al., 2003 [57]	Review	--	--	--	Up to 60% of TBI patients are affected by neuropsychiatric sequelae. Drugs exist to treat specific conditions but RCTs are needed to delineate true treatment effects following TBI.	Anxiety, Cognition, Depression
Lombardi et al., 2008 [58]	Review	--	--	--	Symptomatic treatment studies that understand and address underlying neurobiological recovery processes are needed.	Cognition, PCS
Silver et al., 2009 [59]	Review	--	--	--	Depression and cognitive impairment are common neuropsychiatric symptoms after TBI. Several small studies suggest that SSRIs and tricyclic antidepressants may improve depression in this population.	Cognition, Depression
Tenovuo et al., 2006 [60]	Review	--	--	--	Lack of large scale RCTs place burden on clinician for pharmacologic treatment of TBI-related depression.	Cognition
Wheaton et al., 2011 [61]	Review	--	--	--	Pharmacological treatments that are administered to adults in the postacute stage (≥4 weeks) after TBI have the potential to reduce persistent cognitive and behavioral problems. Sertraline can possibly impair cognition and psychomotor speed.	Cognition
Zafonte et al., 2002 [23]	Review	--	--	--	Limited studies exist among patients with TBI, but serotonin agents including SSRIs seem to be effective for a variety of behavioral disorders. Care should be used when combining agents, and rapid withdrawal should be avoided.	Anxiety, Cognition, Depression

ABS = Agitated Behavioral Scale; AMT = attentional motor task; BDI-II = Beck Depression Inventory, Second Edition; BPRS = Brief Psychiatric Rating Scale; ESS = Epworth Sleepiness Scale; GCS = Glasgow Coma Scale; Ham-D = Hamilton Depression Rating Scale; GOAT = Galveston Orientation and Amnesia Test; MDD = major depressive disorder; MINI = Mini-International Neuropsychiatric Interview; MMSE = Mini-Mental State Examination; MVA = motor vehicle accident; NFI = Neurobehavioral Functioning Inventory; OCD = Obsessive Compulsive Disorder; OL = Orientation Log; PHQ-9 = Patient Health Questionnaire-9; RPQ = Rivermead Post-Concussional Symptoms Questionnaire; SNP = single nucleotide polymorphism; SSRI = selective serotonin reuptake inhibitor; TBI = traumatic brain injury; TCA = tricyclic antidepressant; TMT = Trailmaking Test; USCREMT = University of Southern California Repeatable Episodic Memory Test; WAIS-III = Wechsler Adult Intelligence Test, Third Edition; WMS = Wechsler Memory Scale.

## References

[1] Hyder A.A., Wunderlich C.A., Puvanachandra P., Gururaj G., Kobusingye O.C. (2007). The impact of traumatic brain injuries: A global perspective. NeuroRehabilitation.

[2] Centers for Disease Control and Prevention (2015). Report to Congress on Traumatic Brain Injury in the United States: Epidemiology and Rehabilitation.

[3] Corrigan J.D., Selassie A.W., Orman J.A.L. (2010). The Epidemiology of Traumatic Brain Injury. J. Head Trauma Rehabil..

[4] Bruns J., Hauser W.A. (2003). The epidemiology of traumatic brain injury: A review. Epilepsia.

[5] Kay T., Harrington D.E., Adams R., Anderson T., Berrol S., Cicerone K., Dahlberg C., Gerber D., Goka R., Harley P., Hilt J. (1993). Definition of mild traumatic brain injury. J. Head Trauma Rehabil..

[6] Elgmark Andersson E., Emanuelson I., Björklund R., Stålhammar D.A. (2007). Mild traumatic brain injuries: The impact of early intervention on late sequelae. A randomized controlled trial. Acta Neurochir..

[7] Rutherford W.H., Merrett J.D., McDonald J.R. (1979). Symptoms at one year following concussion from minor head injuries. Injury.

[8] Zaloshnja E., Miller T., Langlois J.A., Selassie A.W. (2008). Prevalence of long-term disability from traumatic brain injury in the civilian population of the United States, 2005. J. Head Trauma Rehabil..

[9] Koponen S., Taiminen T., Portin R., Himanen L., Isoniemi H., Heinonen H., Hinkka S., Tenovuo O. (2002). Axis I and II psychiatric disorders after traumatic brain injury: A 30-year follow-up study. Am. J. Psychiatry.

[10] Koponen S., Taiminen T., Hiekkanen H., Tenovuo O. (2011). Axis I and II psychiatric disorders in patients with traumatic brain injury: A 12-month follow-up study. Brain Inj..

[11] Jacobs B.L., Fornal C.A. (2010). Activity of Brain Serotonergic Neurons in Relation to Physiology and Behavior. Handbook of Behavioral Neuroscience.

[12] Sukonick D.L., Pollock B.G., Sweet R.A., Mulsant B.H., Rosen J., Klunk W.E., Kastango K.B., DeKosky S.T., Ferrell R.E. (2001). The 5-HTTPR*S/*L polymorphism and aggressive behavior in Alzheimer disease. Arch. Neurol..

[13] Zanardi R., Serretti A., Rossini D., Franchini L., Cusin C., Lattuada E., Dotoli D., Smeraldi E. (2001). Factors affecting fluvoxamine antidepressant activity: Influence of pindolol and 5-HTTLPR in delusional and nondelusional depression. Biol. Psychiatry.

[14] Markianos M., Seretis A., Kotsou A., Christopoulos M. (1996). CSF neurotransmitter metabolites in comatose head injury patients during changes in their clinical state. Acta Neurochir..

[15] Tsuiki K., Yamamoto Y.L., Diksic M. (1995). Effect of acute fluoxetine treatment on the brain serotonin synthesis as measured by the alpha-methyl-L-tryptophan autoradiographic method. J. Neurochem..

[16] Tsuiki K., Takada A., Nagahiro S., Grdiša M., Diksic M., Pappius H.M. (1995). Synthesis of Serotonin in Traumatized Rat Brain. J. Neurochem..

[17] Kline A.E., Yu J., Horváth E., Marion D.W., Dixon C.E. (2001). The selective 5-HT(1A) receptor agonist repinotan HCl attenuates histopathology and spatial learning deficits following traumatic brain injury in rats. Neuroscience.

[18] Tiraboschi E., Tardito D., Kasahara J., Moraschi S., Pruneri P., Gennarelli M., Racagni G., Popoli M. (2004). Selective phosphorylation of nuclear CREB by fluoxetine is linked to activation of CaM kinase IV and MAP kinase cascades. Neuropsychopharmacology.

[19] Mattson M.P., Maudsley S., Martin B. (2004). BDNF and 5-HT: A dynamic duo in age-related neuronal plasticity and neurodegenerative disorders. Trends Neurosci..

[20] Kong E.K., Peng L., Chen Y., Yu A.C., Hertz L. (2002). Up-regulation of 5-HT2B receptor density and receptor-mediated glycogenolysis in mouse astrocytes by long-term fluoxetine administration. Neurochem. Res..

[21] Nahon E., Israelson A., Abu-Hamad S., Varda S.B. (2005). Fluoxetine (Prozac) interaction with the mitochondrial voltage-dependent anion channel and protection against apoptotic cell death. FEBS Lett..

[22] Deák F., Lasztóczi B., Pacher P., Petheö G.L., Kecskeméti V., Spät A. (2000). Inhibition of voltage-gated calcium channels by fluoxetine in rat hippocampal pyramidal cells. Neuropharmacology.

[23] Zafonte R.D., Cullen N., Lexell J. (2002). Serotonin agents in the treatment of acquired brain injury. J. Head Trauma Rehabil..

[24] Mostert J.P., Koch M.W., Heerings M., Heersema D.J., De Keyser J. (2008). Therapeutic potential of fluoxetine in neurological disorders. CNS Neurosci. Ther..

[25] Hamilton M. (1960). A rating scale for depression. J. Neurol. Neurosurg. Psychiatry.

[26] Ansari A., Jain A., Sharma A., Mittal R.S., Gupta I.D. (2014). Role of sertraline in posttraumatic brain injury depression and quality-of-life in TBI. Asian J. Neurosurg..

[27] Ashman T.A., Cantor J.B., Gordon W.A., Spielman L., Flanagan S., Ginsberg A., Engmann C., Egan M., Ambrose F., Greenwald B. (2009). A randomized controlled trial of sertraline for the treatment of depression in persons with traumatic brain injury. Arch. Phys. Med. Rehabil..

[28] Baños J.H., Novack T.A., Brunner R., Renfroe S., Lin H.-Y., Meythaler J. (2010). Impact of early administration of sertraline on cognitive and behavioral recovery in the first year after moderate to severe traumatic brain injury. J. Head Trauma Rehabil..

[29] Jorge R.E., Acion L., Burin D.I., Robinson R.G. (2016). Sertraline for Preventing Mood Disorders Following Traumatic Brain Injury: A Randomized Clinical Trial. JAMA Psychiatry.

[30] Lee H., Kim S.-W., Kim J.-M., Shin I.-S., Yang S.-J., Yoon J.-S. (2005). Comparing effects of methylphenidate, sertraline and placebo on neuropsychiatric sequelae in patients with traumatic brain injury. Hum. Psychopharmacol..

[31] Meythaler J.M., Depalma L., Devivo M.J., Guin-Renfroe S., Novack T.A. (2001). Sertraline to improve arousal and alertness in severe traumatic brain injury secondary to motor vehicle crashes. Brain Inj..

[32] Novack T.A., Baños J.H., Brunner R., Renfroe S., Meythaler J.M. (2009). Impact of early administration of sertraline on depressive symptoms in the first year after traumatic brain injury. J. Neurotrauma.

[33] Rapoport M.J., Mitchell R.A., McCullagh S., Herrmann N., Chan F., Kiss A., Feinstein A., Lanctôt K.L. (2010). A randomized controlled trial of antidepressant continuation for major depression following traumatic brain injury. J. Clin. Psychiatry.

[34] Dolberg O.T., Klag E., Gross Y., Schreiber S. (2002). Relief of serotonin selective reuptake inhibitor induced sexual dysfunction with low-dose mianserin in patients with traumatic brain injury. Psychopharmacology.

[35] Fann J.R., Uomoto J.M., Katon W.J. (2000). Sertraline in the treatment of major depression following mild traumatic brain injury. J. Neuropsychiatry Clin. Neurosci..

[36] Horsfield S.A., Rosse R.B., Tomasino V., Schwartz B.L., Mastropaolo J., Deutsch S.I. (2002). Fluoxetine’s effects on cognitive performance in patients with traumatic brain injury. Int. J. Psychiatry Med..

[37] Lanctôt K.L., Rapoport M.J., Chan F., Rajaram R.D., Strauss J., Sicard T., McCullagh S., Feinstein A., Kiss A., Kennedy J.L. (2010). Genetic predictors of response to treatment with citalopram in depression secondary to traumatic brain injury. Brain Inj..

[38] Luo L., Chai Y., Jiang R., Chen X., Yan T. (2015). Cortisol Supplement Combined with Psychotherapy and Citalopram Improves Depression Outcomes in Patients with Hypocortisolism after Traumatic Brain Injury. Aging Dis..

[39] Muller U., Murai T., Bauer-Wittmund T., von Cramon D.Y. (1999). Paroxetine versus citalopram treatment for pathological crying after brain injury. Brain Inj..

[40] Perino C., Rago R., Cicolini A., Torta R., Monaco F. (2001). Mood and behavioural disorders following traumatic brain injury: Clinical evaluation and pharmacological management. Brain Inj..

[41] Rapoport M.J., Chan F., Lanctot K., Herrmann N., McCullagh S., Feinstein A. (2008). An open-label study of citalopram for major depression following traumatic brain injury. J. Psychopharmacol..

[42] Turner-Stokes L., Hassan N., Pierce K., Clegg F. (2002). Managing depression in brain injury rehabilitation: The use of an integrated care pathway and preliminary report of response to sertraline. Clin. Rehabil..

[43] Hensley P.L., Reeve A. (2001). A case of antidepressant-induced akathisia in a patient with traumatic brain injury. J. Head Trauma Rehabil..

[44] Nahas Z., Arlinghaus K.A., Kotrla K.J., Clearman R.R., George M.S. (1998). Rapid response of emotional incontinence to selective serotonin reuptake inhibitors. J. Neuropsychiatry Clin. Neurosci..

[45] Patterson D.E., Braverman S.E., Belandres P.V. (1997). Speech dysfunction due to trazodone--fluoxetine combination in traumatic brain injury. Brain Inj..

[46] Scheutzow M.H., Wiercisiewski D.R. (1999). Panic disorder in a patient with traumatic brain injury: A case report and discussion. Brain Inj..

[47] Slaughter J., Bobo W., Childers M.K. (1999). Selective serotonin reuptake inhibitor treatment of post-traumatic Klüver-Bucy syndrome. Brain Inj..

[48] Sloan R.L., Brown K.W., Pentland B. (1992). Fluoxetine as a treatment for emotional lability after brain injury. Brain Inj..

[49] Spinella M., Eaton L.A. (2002). Hypomania induced by herbal and pharmaceutical psychotropic medicines following mild traumatic brain injury. Brain Inj..

[50] Stanislav S.W., Childs N.L. (1999). Dystonia associated with sertraline. J. Clin. Psychopharmacol..

[51] Stengler-Wenzke K., Müller U. (2002). Fluoxetine for OCD after brain injury. Am. J. Psychiatry.

[52] Workman E.A., Harrington D.P. (1992). Sertraline-augmented lithium therapy of organic mood syndrome. Psychosomatics.

[53] Wroblewski B.A., Guidos A., Leary J., Joseph A.B. (1992). Control of depression with fluoxetine and antiseizure medication in a brain-injured patient. Am. J. Psychiatry.

[54] Fann J.R., Hart T., Schomer K.G. (2009). Treatment for depression after traumatic brain injury: A systematic review. J. Neurotrauma.

[55] Fleminger S., Oliver D.L., Williams W.H., Evans J. (2003). The neuropsychiatry of depression after brain injury. Neuropsychol. Rehabil..

[56] Jorge R., Robinson R.G. (2003). Mood disorders following traumatic brain injury. Int. Rev. Psychiatry.

[57] Lee H.B., Lyketsos C.G., Rao V. (2003). Pharmacological management of the psychiatric aspects of traumatic brain injury. Int. Rev. Psychiatry.

[58] Lombardi F. (2008). Pharmacological treatment of neurobehavioural sequelae of traumatic brain injury. Eur. J. Anaesthesiol. Suppl..

[59] Silver J.M., McAllister T.W., Arciniegas D.B. (2009). Depression and Cognitive Complaints Following Mild Traumatic Brain Injury. Am. J. Psychiatry.

[60] Tenovuo O. (2006). Pharmacological enhancement of cognitive and behavioral deficits after traumatic brain injury. Curr. Opin. Neurol..

[61] Wheaton P., Mathias J.L., Vink R. (2011). Impact of pharmacological treatments on cognitive and behavioral outcome in the postacute stages of adult traumatic brain injury: A meta-analysis. J. Clin. Psychopharmacol..

[62] Hamilton W.K., Aydin B., Mizumoto A. (2015). MAVIS: Meta Analysis via Shiny.

[63] Cooper H., Hedges L.V., Valentine J.C. (2009). The Handbook of Research Synthesis and Meta-Analysis.

[64] Albert P.R., Benkelfat C. (2013). The neurobiology of depression—Revisiting the serotonin hypothesis. II. Genetic, epigenetic and clinical studies. Philos. Trans. R. Soc. Lond B Biol. Sci..

[65] McAllister T.W. (2011). Neurobiological consequences of traumatic brain injury. Dialogues Clin. Neurosci..

[66] Bombardier C.H., Fann J.R., Temkin N.R., Esselman P.C., Barber J., Dikmen S.S. (2010). Rates of major depressive disorder and clinical outcomes following traumatic brain injury. JAMA.

[67] Keller M.B., Kocsis J.H., Thase M.E., Gelenberg A.J., Rush A.J., Koran L., Schatzberg A., Russell J., Hirschfeld R., Klein D. (1998). Maintenance phase efficacy of sertraline for chronic depression: A randomized controlled trial. JAMA.

[68] Kocsis J.H., Schatzberg A., Rush A.J., Klein D.N., Howland R., Gniwesch L., Davis S.M., Harrison W. (2002). Psychosocial outcomes following long-term, double-blind treatment of chronic depression with sertraline vs. placebo. Arch. Gen. Psychiatry.

[69] Sullivan P.F., Neale M.C., Kendler K.S. (2000). Genetic epidemiology of major depression: Review and meta-analysis. Am. J. Psychiatry.

[70] Capruso D.X., Levin H.S. (1992). Cognitive impairment following closed head injury. Neurol. Clin..

[71] Elder G.A. (2015). Update on TBI and Cognitive Impairment in Military Veterans. Curr. Neurol. Neurosci. Rep..

[72] Cullen B., O’Neill B., Evans J.J., Coen R.F., Lawlor B.A. (2007). A review of screening tests for cognitive impairment. J. Neurol. Neurosurg. Psychiatry.

[73] Hibbard M.R., Ashman T.A., Spielman L.A., Chun D., Charatz H.J., Melvin S. (2004). Relationship between depression and psychosocial functioning after traumatic brain injury. Arch. Phys. Med. Rehabil..

[74] Resch J.A., Villarreal V., Johnson C.L., Elliott T.R., Kwok O.-M., Berry J.W., Underhill A.T. (2009). Trajectories of life satisfaction in the first 5 years following traumatic brain injury. Rehabil. Psychol..

[75] Fann J.R., Uomoto J.M., Katon W.J. (2001). Cognitive improvement with treatment of depression following mild traumatic brain injury. Psychosomatics.

[76] Santarelli L., Saxe M., Gross C., Surget A., Battaglia F., Dulawa S., Weisstaub N., Lee J., Duman R., Arancio O. (2003). Requirement of hippocampal neurogenesis for the behavioral effects of antidepressants. Science.

[77] Malberg J.E., Eisch A.J., Nestler E.J., Duman R.S. (2000). Chronic antidepressant treatment increases neurogenesis in adult rat hippocampus. J. Neurosci..

[78] Wang J.W., David D.J., Monckton J.E., Battaglia F., Hen R. (2008). Chronic fluoxetine stimulates maturation and synaptic plasticity of adult-born hippocampal granule cells. J. Neurosci..

[79] Naudon L., Hotte M., Jay T.M. (2007). Effects of acute and chronic antidepressant treatments on memory performance: A comparison between paroxetine and imipramine. Psychopharmacology.

[80] Vythilingam M., Vermetten E., Anderson G.M., Luckenbaugh D., Anderson E.R., Snow J., Staib L.H., Charney D.S., Bremner J.D. (2004). Hippocampal volume, memory, and cortisol status in major depressive disorder: Effects of treatment. Biol. Psychiatry.

[81] Gasquione P.G. (1997). Postconcussion symptoms. Neuropsychol. Rev..

[82] Evans R.W. (1992). The postconcussion syndrome and the sequelae of mild head injury. Neurol. Clin..

[83] Ryan L.M., Warden D.L. (2003). Post concussion syndrome. Int. Rev. Psychiatry.

[84] Arciniegas D.B., Anderson C.A., Topkoff J., McAllister T.W. (2005). Mild traumatic brain injury: A neuropsychiatric approach to diagnosis, evaluation, and treatment. Neuropsychiatr. Dis. Treat..

[85] Scher L.M., Loomis E., McCarron R.M. (2011). Traumatic brain injury: Pharmacotherapy options for cognitive deficits. Curr. Psychiatry.

[86] King N.S., Crawford S., Wenden F.J., Moss N.E., Wade D.T. (1995). The Rivermead Post Concussion Symptoms Questionnaire: A measure of symptoms commonly experienced after head injury and its reliability. J. Neurol..

[87] Babcock L., Byczkowski T., Wade S.L., Ho M., Mookerjee S., Bazarian J.J. (2013). Predicting postconcussion syndrome after mild traumatic brain injury in children and adolescents who present to the emergency department. JAMA Pediatr..

[88] Ingebrigtsen T., Waterloo K., Marup-Jensen S., Attner E., Romner B. (1998). Quantification of post-concussion symptoms 3 months after minor head injury in 100 consecutive patients. J. Neurol..

[89] Potter S., Leigh E., Wade D., Fleminger S. (2006). The Rivermead Post Concussion Symptoms Questionnaire: A confirmatory factor analysis. J. Neurol..

[90] Eyres S., Carey A., Gilworth G., Neumann V., Tennant A. (2005). Construct validity and reliability of the Rivermead Post-Concussion Symptoms Questionnaire. Clin. Rehabil..

[91] Kapur S., Remington G. (1996). Serotonin-dopamine interaction and its relevance to schizophrenia. Am. J. Psychiatry.

[92] Ouellet M.-C., Savard J., Morin C.M. (2004). Book Review: Insomnia following Traumatic Brain Injury: A Review. Neurorehabil. Neural Repair.

[93] Viola-Saltzman M., Watson N.F. (2012). Traumatic brain injury and sleep disorders. Neurol. Clin..

[94] Srinivasan V., Pandi-Perumal S.R., Trahkt I., Spence D.W., Poeggeler B., Hardeland R., Cardinali D.P. (2009). Melatonin and melatonergic drugs on sleep: Possible mechanisms of action. Int. J. Neurosci..

[95] Beasley C.M., Koke S.C., Nilsson M.E., Gonzales J.S. (2000). Adverse events and treatment discontinuations in clinical trials of fluoxetine in major depressive disorder: An updated meta-analysis. Clin. Ther..

[96] Yıldız A., Gönül A.S., Tamam L. (2002). Mechanism of actions of antidepressants: Beyond the receptors. Bull. Clin. Psychopharmacol..

[97] Portas C.M., Bjorvatn B., Ursin R. (2000). Serotonin and the sleep/wake cycle: Special emphasis on microdialysis studies. Prog. Neurobiol..

[98] Scahill L., Carroll D., Burke K. (2004). Methylphenidate: Mechanism of action and clinical update. J. Child Adolesc. Psychiatr. Nurs..

[99] Sobanski E., Schredl M., Kettler N., Alm B. (2008). Sleep in adults with attention deficit hyperactivity disorder (ADHD) before and during treatment with methylphenidate: A controlled polysomnographic study. Sleep.

[100] Ashman T.A., Spielman L.A., Hibbard M.R., Silver J.M., Chandna T., Gordon W.A. (2004). Psychiatric challenges in the first 6 years after traumatic brain injury: Cross-sequential analyses of Axis I disorders. Arch. Phys. Med. Rehabil..

[101] Deb S., Lyons I., Koutzoukis C., Ali I., McCarthy G. (1999). Rate of psychiatric illness 1 year after traumatic brain injury. Am. J. Psychiatry.

[102] Bryant R.A., O’Donnell M.L., Creamer M., McFarlane A.C., Clark C.R., Silove D. (2010). The psychiatric sequelae of traumatic injury. Am. J. Psychiatry.

[103] Soomro G.M., Altman D., Rajagopal S., Oakley-Browne M. (2008). Selective serotonin re-uptake inhibitors (SSRIs) versus placebo for obsessive compulsive disorder (OCD). Cochrane Database Syst. Rev..

[104] Thomsen P.H. (2000). Obsessive-compulsive disorder: Pharmacological treatment. Eur. Child Adolesc. Psychiatry 9.

[105] Van Reekum R., Cohen T., Wong J. (2000). Can traumatic brain injury cause psychiatric disorders?. J. Neuropsychiatry Clin. Neurosci..

[106] Kanetani K., Kimura M., Endo S. (2003). Therapeutic effects of milnacipran (serotonin noradrenalin reuptake inhibitor) on depression following mild and moderate traumatic brain injury. J. Nippon Med. Sch..

[107] Lane R.M. (1998). SSRI-induced extrapyramidal side-effects and akathisia: Implications for treatment. J. Psychopharmacol..

[108] Prabhakar D., Balon R. (2010). How do SSRIs cause sexual dysfunction? Understanding key mechanisms can help improve patient adherence, prognosis. Curr. Psychiatry.

[109] Howlett J.R., Stein M.B., Laskowitz D., Grant G. (2015). Post-Traumatic Stress Disorder: Relationship to Traumatic Brain Injury and Approach to Treatment. Translational Research in Traumatic Brain Injury.

[110] Stein M.B., McAllister T.W. (2009). Exploring the Convergence of Posttraumatic Stress Disorder and Mild Traumatic Brain Injury. Am. J. Psychiatry.

[111] Lew H.L., Vanderploeg R.D., Moore D.F., Schwab K., Friedman L., Yesavage J., Keane T.M., Warden D.L., Sigford B.J. (2008). Overlap of mild TBI and mental health conditions in returning OIF/OEF service members and veterans. J. Rehabil. Res. Dev..

[112] Marshall R.D., Beebe K.L., Oldham M., Zaninelli R. (2001). Efficacy and safety of paroxetine treatment for chronic PTSD: A fixed-dose, placebo-controlled study. Am. J. Psychiatry.

[113] Brady K., Pearlstein T., Asnis G.M., Baker D., Rothbaum B., Sikes C.R., Farfel G.M. (2000). Efficacy and safety of sertraline treatment of posttraumatic stress disorder: A randomized controlled trial. JAMA.

[114] Connor K.M., Sutherland S.M., Tupler L.A., Malik M.L., Davidson J.R. (1999). Fluoxetine in post-traumatic stress disorder. Randomised, double-blind study. Br. J. Psychiatry.

[115] Raskind M.A., Peskind E.R., Hoff D.J., Hart K.L., Holmes H.A., Warren D., Shofer J., O’Connell J., Taylor F., Gross C. (2007). A parallel group placebo controlled study of prazosin for trauma nightmares and sleep disturbance in combat veterans with post-traumatic stress disorder. Biol. Psychiatry.

[116] Whyte J., Vaccaro M., Grieb-Neff P., Hart T. (2002). Psychostimulant use in the rehabilitation of individuals with traumatic brain injury. J. Head Trauma Rehabil..

[117] Meythaler J.M., Brunner R.C., Johnson A., Novack T.A. (2002). Amantadine to improve neurorecovery in traumatic brain injury-associated diffuse axonal injury: A pilot double-blind randomized trial. J. Head Trauma Rehabil..

[118] Reinhard D.L., Whyte J., Sandel M.E. (1996). Improved arousal and initiation following tricyclic antidepressant use in severe brain injury. Arch. Phys. Med. Rehabil..

[119] Van Waes V., Beverley J., Marinelli M., Steiner H. (2010). Selective serotonin reuptake inhibitor antidepressants potentiate methylphenidate (Ritalin)-induced gene regulation in the adolescent striatum. Eur. J. Neurosci..

